# Older Adults in Poor Health Received Social Support Mainly From Family: The Singapore Chinese Health Study

**DOI:** 10.1093/geroni/igaa057.548

**Published:** 2020-12-16

**Authors:** Jon Barrenetxea, Yang Yi, Kyriakos S Markides, Woon Puay Koh, Feng Qiushi

**Affiliations:** 1 Duke-NUS Medical School, Singapore, Singapore; 2 The University of Texas Medical Branch at Galveston, Galveston, United States; 3 National University of Singapore, Singapore, Singapore

## Abstract

While having social support can contribute to the health of older adults, those in poor health may be limited in their capacity to receive social support. We studied health factors associated with social support among 16,948 participants from follow-up 3 of the Singapore Chinese Health Study, a population-based cohort of older Singapore Chinese. Participants were interviewed at mean age of 73 years (range from 61 to 96 years) using the Duke Social Support Scale. Latent Class Analysis (LCA) was applied to derive groups based on the source and intensity of social support. We ran multivariate logistic regression models to study health factors associated with group membership. LCA revealed four groups in increasing social support scores: The “family restricted”, who had the lowest social support scores and only received support from family (50%); the “loners”, who had some support from extended family and non-family (5%); the “family oriented”, who had broad family support and some non-family support (28%); and the “overall supported”, who had the highest social support scores and received broad support from family, extended family and non-family (17%). Compared to the “overall supported” group, health factors associated with being “family restricted” were: having instrumental limitations [odds ratio (OR) 1.34, 95% confidence interval (CI) 1.19-1.50], having poor self-rated health (OR 1.40, 95% CI 1.28-1.54), being depressed (OR 2.49, 95% CI 2.21-2.81) and being cognitively impaired (OR 1.19, 95% CI 1.04-1.37). Our results showed that older adults in poor health received social support mainly from family.

